# Ensemble biomarkers for guiding anti‐angiogenesis therapy for ovarian cancer using deep learning

**DOI:** 10.1002/ctm2.1162

**Published:** 2023-01-13

**Authors:** Ching‐Wei Wang, Yu‐Ching Lee, Yi‐Jia Lin, Chun‐Chieh Chang, Aung‐Kyaw‐Oo Sai, Chih‐Hung Wang, Tai‐Kuang Chao

**Affiliations:** ^1^ Graduate Institute of Biomedical Engineering National Taiwan University of Science and Technology Taipei Taiwan; ^2^ Graduate Institute of Applied Science and Technology National Taiwan University of Science and Technology Taipei Taiwan; ^3^ Department of Pathology Tri‐Service General Hospital Taipei Taiwan; ^4^ Institute of Pathology and Parasitology National Defense Medical Center Taipei Taiwan; ^5^ Department of Otolaryngology‐Head and Neck Surgery Tri‐Service General Hospital Taipei Taiwan; ^6^ Department of Otolaryngology‐Head and Neck Surgery National Defense Medical Center Taipei Taiwan

Dear Editor,

The central contribution of this research is to present highly effective ensemble biomarkers for guiding therapies to ovarian cancer, which has become the major cause of death from gynecologic cancer in industrialized countries^1^ whereas 90% of all ovarian cancer types is epithelial ovarian cancer (EOC). The typical therapy for EOC is a combination of cytoreductive surgery, platinum and taxane chemotherapy.^2^, ^3^ Bevacizumab (Avastin), which is the first FDA approved anti‐angiogenic agent,^4^, ^5^ leads to normalization of tumor vasculature and improved effectiveness of standard therapy with promising result, but unfortunately it is associated with high degree of adverse effects, including arterial thromboembolism, high blood pressure, presence of excess proteins in the urine, haemorrhage, poor wound healing and ruptured bowel.^6^ The goal of this study is to examine whether the angiogenesis‐related biomarkers, such as VEGF, Ang‐2 and PKM2 could be used to build an effective therapeutic predictive system for patient selection, guiding ovarian cancer treatment. In this study, we investigate three angiogenesis‐related potential biomarkers, including VEGF, Angiopoietin 2 (Ang‐2) and Pyruvate kinase isoform M2 (PKM2), and develop an annotation‐free instance boosting deep learning ensemble framework to forecast the treatment response to bevacizumab of patients with EOC or peritoneal serous papillary carcinoma (PSPC) using tissue microarrays. See Supporting Information (Section 1) for related works.

To enable the development of therapeutic predictive AI systems, digitized whole slide images (WSIs) of tissue microarrays are collected from samples with EOC and PSPC, containing 720 female patient sample tissue cores in total. For the data inclusion and exclusion criteria, patients are eligible for the retrospective study if they are EOC or PSPC patients receiving 1) concurrent bevacizumab therapy, 2) second‐line bevacizumab therapy post recurrence or 3) maintenance bevacizumab therapy. On the other hand, patients are ineligible if they are borderline malignant tumor or not primary ovarian or retroperitoneal carcinoma. The data include papillary serous carcinoma (444 samples), clear cell carcinoma (69 samples), endometrioid carcinoma (39 samples), mucinous carcinoma (10 samples), unclassified carcinoma (69 samples), and PSPC (89 samples) of 12 TMAs. This study has been approved by the hospital research ethics committee with the IRB approval number (TSGHIRB No.1‐107‐05‐171 and No. B202005070), and we construct the database and trace patient data from the period of 2013/3 to 2021/1. Supplemental Table S0(a) presents the baseline characteristics of the database constructed in this study. The flowchart for training individual treatment effectiveness classifiers of the proposed method is illustrated in Figure 1, and the decision inference workflow of the proposed ensemble framework is illustrated in Figure 2. See Supporting Information (Section 2‐1) for more data information and (Section 2‐2) for the proposed method, respectively.

**FIGURE 1:**
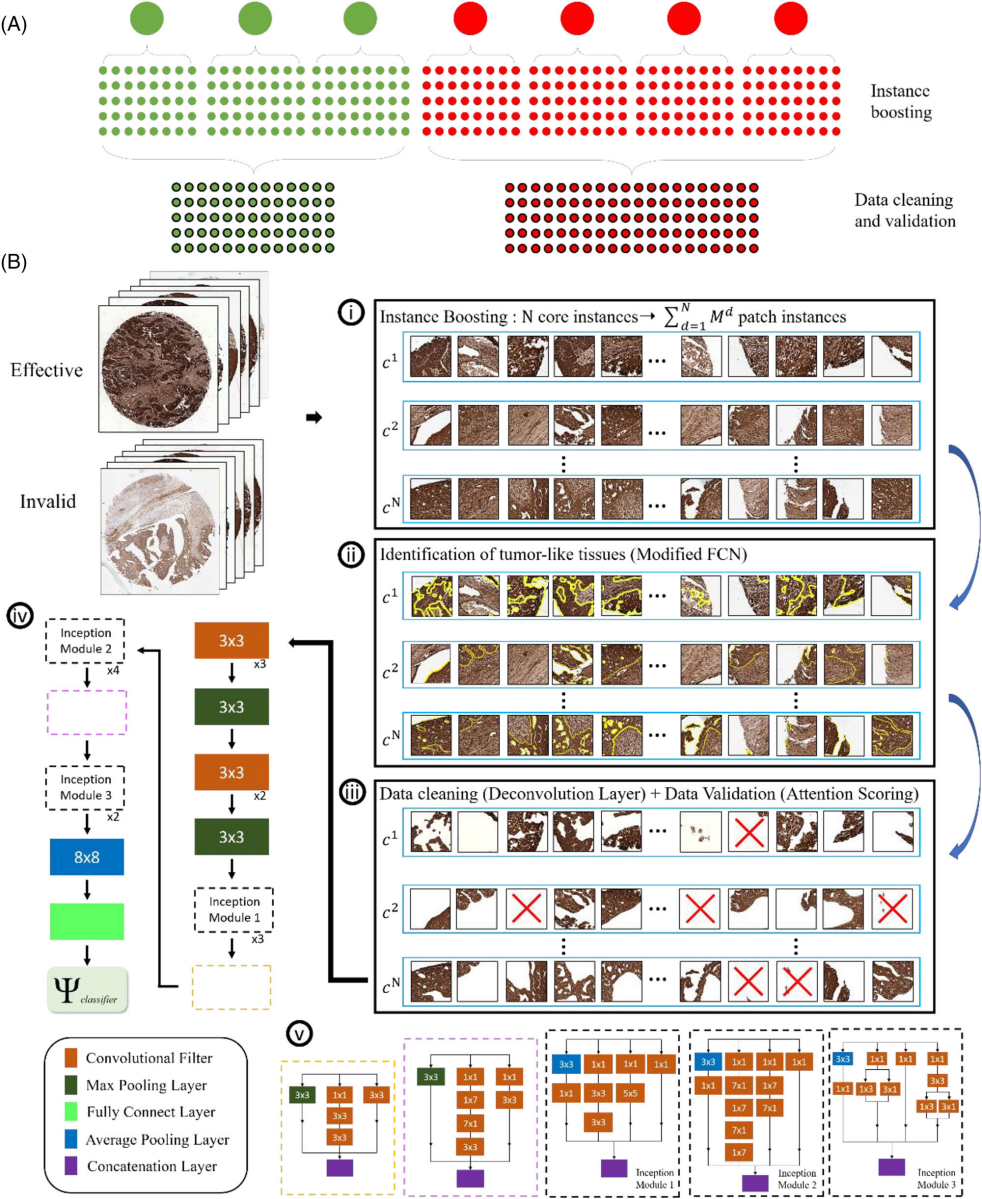
The flowchart for training individual treatment effectiveness classifiers of the proposed method. (A) Firstly each tissue core instance is split into multiple patch unit instances, which enables utilization of detailed high resolution data for computational efficiency and greatly increase the number training instance, and secondly a large and clean training database with detailed useful information is produced by the proposed data cleaning and validation module. (B.i‐iii) Sample results of instance boosting, localization of important tumor‐like information, data cleaning and validation processes. (B.iv) The network architecture of the treatment effectiveness prediction model using inception V3 with (B.v) dimensional reduction and parallel structures of the inception modules

**FIGURE 2:**
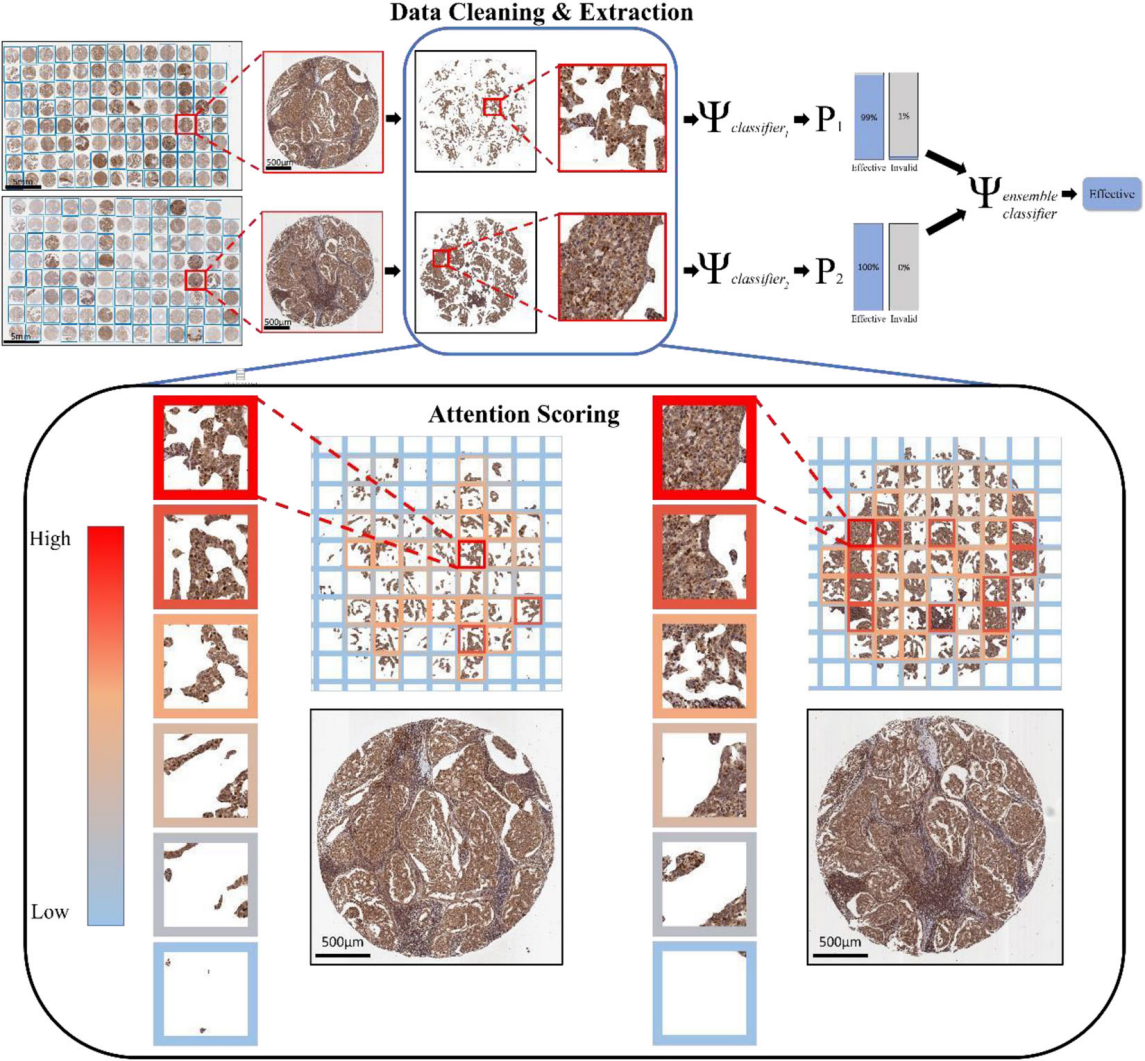
The decision inference workflow of the proposed ensemble framework. Individual tissue cores are rapidly located by the core detector in the low magnification level. Data of interests are identified by the weakly supervised tumor selection model and cleaned by the data cleaning module at the high magnification level. Patches are then scored by attention patch scoring, and the patch with the highest score for each staining, containing the richest tumor information, is selected as the input to the pre‐trained classifier to render a probability vector. Multiple probability vectors by individual classifiers are then integrated by the proposed ensemble to render the final prediction

For quantitative evaluation, the experiments were conducted in four parts. Firstly, the whole dataset (with 720 patient sample tissue cores) was randomly sampled and separated into two independent subsets, including 472 cores (66%) for training and 248 cores (34%) for testing as shown in Supplemental Table S0(b). Next, to further validate the effectiveness of individual models' predictions in how patients respond to the treatment, two generally adopted statistical analyses,^7^ including the univariate Kaplan‐Meier survival (K‐M) analysis and the multivariate Cox hazards analysis, were conducted. Fourthly, to examine the generalizability of individual models for unseen data, quantitative evaluation was performed using 5 ‐fold cross validation (CV).

In the first experiment, the proposed biomarker system using PKM2 or Ang‐2 achieves excellent performance in accuracy 98%, precision 100%, recall 97%, F1‐score 99%, and AUC 99.7% (see Table 1a). According to the results using a single biomarker, the top two potential markers were then selected to build a hybrid marker‐based system for further investigation of a possible even better prediction system. The proposed ensemble using both PKM2 and Ang‐2 data achieves perfect prediction results, obtaining 100% in all measurements. Furthermore, Figure 3A compares the ROC curves of individual biomarker systems, and the proposed ensemble biomarker system provides the best treatment effectiveness classification accuracy, invariant to the threshold value. Secondly, Cox hazards analysis confirms that the proposed ensemble model is statistically significantly effective (*p* = 0.012), and (HR = 0.23) indicates that patients who are predicted with effective treatment responses are 0.23 times as likely to get cancer recurrence, compared to patients predicted with invalid responses (Table 1b). Thirdly, K‐M analysis shows that there are very strong evidences that the proposed ensemble using both PKM2 and Ang‐2 and the proposed models using PKM2 or Ang‐2 are capable of identifying patients acquiring favorable treatment effects with low risk of recurrent cancer in the progression free survival time with high statistical significance (p<0.001) (Figure 3B). See detailed evaluation and statistical analysis in Supporting Information (Section 3).

**TABLE 1a:** Evaluation in classification of therapeutic outcomes (66% training, 34% testing)

Method	Data	Accuracy	Precision	Recall	F‐measure	AUC	Group Rank (F)	Overall Rank (F)
Wang et al.^10^	PKM2	0.63	0.62	0.89	0.73	0.590	4	13
Campanella et al.^8^	PKM2	0.92	0.92	0.94	0.93	0.972	2	5
Coudray et al.^9^	PKM2	0.66	0.63	0.91	0.77	0.965	3	10
Proposed Method	PKM2	**0.98**	**1**	**0.97**	**0.99**	**0.997**	1	2
Wang et al.^10^	Ang‐2	0.71	0.76	0.71	0.74	0.680	4	12
Campanella et al.^8^	Ang‐2	0.89	0.89	0.91	0.90	0.964	2	6
Coudray et al.^9^	Ang‐2	0.77	0.82	0.77	0.79	0.824	3	8
Proposed Method	Ang‐2	**0.98**	**1**	**0.97**	**0.99**	**0.997**	1	2
Wang et al.^10^	VEGF	0.69	0.70	0.80	0.75	0.689	3	11
Campanella et al.^8^	VEGF	**0.74**	0.73	**0.86**	**0.79**	0.823	1	8
Coudray et al.^9^	VEGF	0.65	0.64	0.83	0.73	0.711	4	8
Proposed Method	VEGF	**0.74**	**0.76**	0.80	0.78	**0.831**	2	9
Wang et al.^10^	PKM2+Ang‐2	0.76	0.73	0.86	0.79	0.857	6	8
Campanella et al.^8^	PKM2+Ang‐2	0.79	0.79	0.86	0.82	0.872	5	7
Coudray et al.^9^	PKM2+Ang‐2	0.94	**1**	0.89	0.94	0.999	2	4
Ensemble + Wang et al.^10^	PKM2+Ang‐2	0.60	0.61	0.80	0.69	0.709	7	14
Ensemble + Campanella et al.^8^	PKM2+Ang‐2	0.95	0.94	0.97	0.96	0.975	3	3
Ensemble + Coudray et al.^9^	PKM2+Ang‐2	0.89	0.87	0.94	0.90	0.966	4	6
Proposed Method	PKM2+Ang‐2	**1**	**1**	**1**	**1**	**1**	1	1

*Note*: In the last group of Table 1, the first three rows represent the result of each model trained based on the concatenation of the images of the two markers, while the fourth to sixth row represent the result of ensemble decisions from two separate models, and each model is trained independently from the images of individual markers.

**FIGURE 3:**
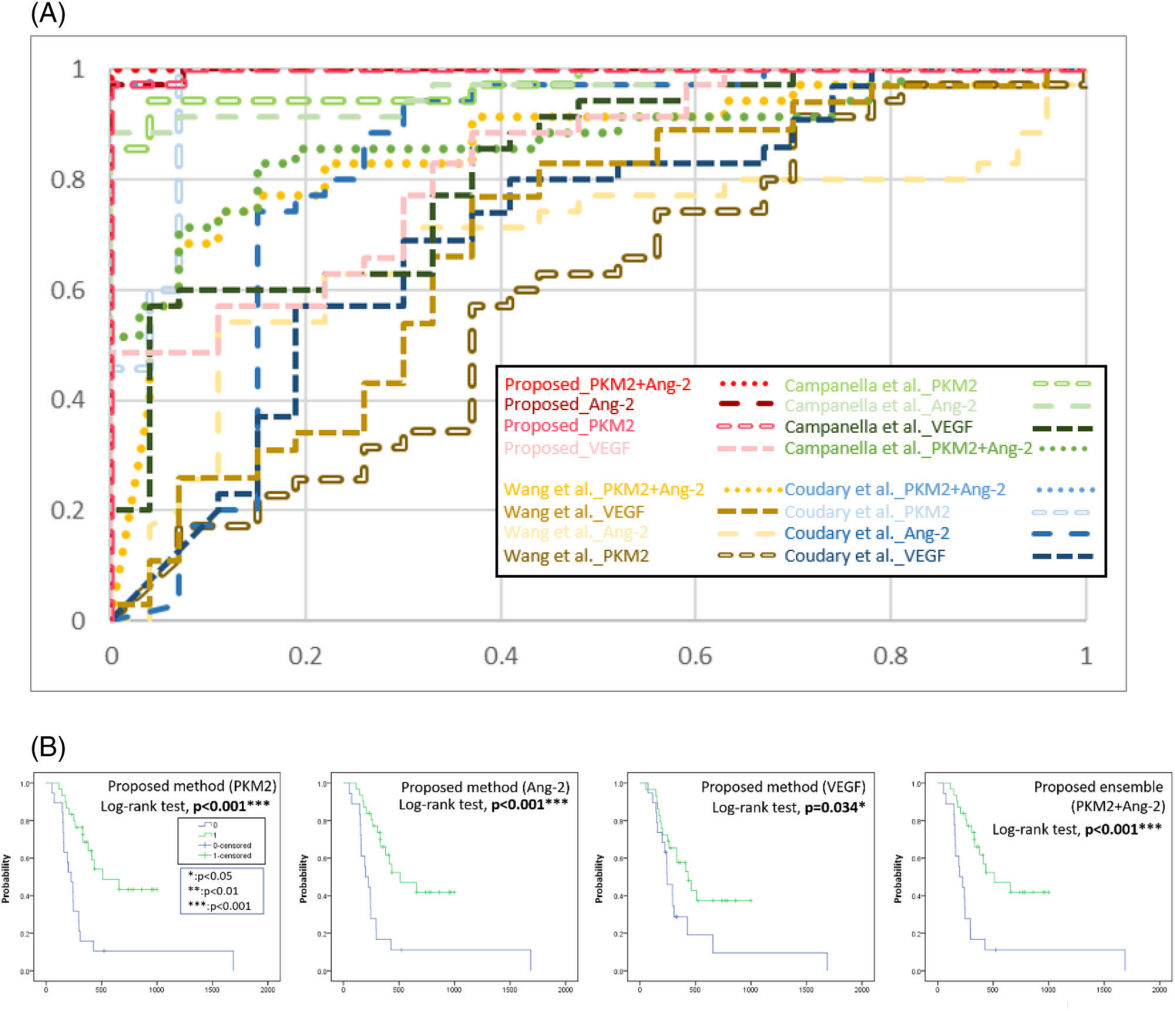
(A) Receiver operating characteristic (ROC) curves of individual biomarker systems. (B) Kaplan–Meier survival analysis based on the treatment response prediction outcomes of the proposed model (0: invalid; 1: effective) with the progression‐free survival time

**TABLE 1b:** Results of cox hazards analysis in cancer recurrence

	Adjusted Hazard Ratio (within 95% Confidence Interval)	P‐value
Age	0.98 (0.94–1.03)	0.403
BMI	0.98 (0.89–1.08)	0.694
Number of bevacizumab used times	0.99 (0.19–1.09)	0.874
FIGO stage (III+IV vs. I+II)II	2.65 (0.57–12.38)	0.216
Histology (others vs. serous)	1.58 (0.55–4.52)	0.395
**Surgery**		
CRS+HIPEC	1.00 (reference)	reference
optimal	0.87 (0.31–2.44)	0.797
suboptimal	0.95 (0.32–2.85)	0.928
**Therapy**		
Concurrent therapy	1.00 (reference)	reference
Second‐line therapy	0.22 (0.04–1.24)	0.086
Maintenance therapy	2.25 (0.71–7.10)	0.165
**Proposed system prediction**		
Ensemble Biomarkers	0.23 (0.08–0.72)	**0.012** *

* Statistical significance (*p*‐value less than 0.05).

Fourthly, to further examine the generalizability of individual models for unseen data, quantitative evaluation was performed based on 5‐fold CV. As shown in Table 1c, even with 5‐fold CV, the proposed ensemble model using both PKM2 and Ang‐2 data consistently achieves near‐to‐perfect prediction in patient response to the treatment, obtaining accuracy 99%±3%, precision 99%±2%, recall 99%±2%, f‐measure 99%±2% and AUC100%±0%. Moreover, the proposed biomarker system based on single PKM2 or Ang‐2 data also obtains high accuracy and precision (0.91±0.05,0.98±0.03 using PKM2); (0.96±0.01,0.95±0.03 using Ang‐2), respectively.

**TABLE 1c:** Evaluation in classification of therapeutic outcomes based on the five‐fold CV

Method	Data	Accuracy	Precision	Recall	F‐measure	AUC	Group Rank (F)	Overall Rank (F)
Wang et al.^10^	PKM2	0.60 ± 0.09	0.63 ± 0.08	0.78 ± 0.19	0.68 ± 0.09	0.64 ± 0.11	4	13
Campanella et al.^8^	PKM2	0.84 ± 0.08	0.87 ± 0.08	0.86 ± 0.13	0.86 ± 0.08	0.92 ± 0.08	2	5
Coudray et al.^9^	PKM2	0.71 ± 0.16	0.72 ± 0.17	**0.89** ± **0.14**	0.78 ± 0.11	0.75 ± 0.23	3	9
Proposed Method	PKM2	**0.91** ± **0.05**	**0.98** ± **0.03**	0.86 ± 0.11	**0.91** ± **0.05**	**0.99** ± **0.01**	1	4
Wang et al.^10^	Ang‐2	0.70 ± 0.12	0.75 ± 0.12	0.74 ± 0.19	0.73 ± 0.11	0.78 ± 0.06	3	12
Campanella et al.^8^	Ang‐2	0.92 ± 0.01	0.94 ± 0.06	0.91 ± 0.04	0.93 ± 0.02	0.98 ± 0.01	2	3
Coudray et al.^9^	Ang‐2	0.64 ± 0.11	0.79 ± 0.17	0.62 ± 0.34	0.60 ± 0.29	0.74 ± 0.13	4	15
Proposed Method	Ang‐2	**0.96** ± **0.01**	**0.95** ± **0.03**	**0.97** ± **0.04**	**0.96** ± **0.01**	**1.00** ± **0.01**	1	2
Wang et al.^10^	VEGF	0.55 ± 0.05	0.59 ± 0.04	0.73 ± 0.14	0.64 ± 0.06	0.58 ± 0.09	3	14
Campanella et al.^8^	VEGF	0.74 ± 0.07	0.81 ± 0.09	0.75 ± 0.17	0.77 ± 0.09	0.75 ± 0.07	2	10
Coudray et al.^9^	VEGF	0.55 ± 0.07	0.68 ± 0.10	0.47 ± 0.33	0.50 ± 0.19	0.60 ± 0.10	4	16
Proposed Method	VEGF	**0.80** ± **0.09**	**0.84** ± **0.11**	**0.83** ± **0.15**	**0.82** ± **0.08**	**0.89** ± **0.08**	1	7
Wang et al.^10^	PKM2+Ang‐2	0.70 ± 0.05	0.74 ± 0.07	0.77 ± 0.17	0.74 ± 0.07	0.88 ± 0	4	11
Campanella et al.^8^	PKM2+Ang‐2	0.81 ± 0.07	0.84 ± 0.11	0.86 ± 0.07	0.84 ± 0.04	0.89 ± 0.06	3	6
Coudray et al.^9^	PKM2+Ang‐2	0.57 ± 0.15	0.51 ± 0.50	0.39 ± 0.43	0.40 ± 0.38	0.60 ± 0.34	7	17
Ensemble + Wang et al.^10^	PKM2+Ang‐2	0.66 ± 0.07	0.67 ± 0.05	0.83 ± 0.17	0.73 ± 0.08	0.76 ± 0.10	6	12
Ensemble + Campanella et al.^8^	PKM2+Ang‐2	0.95 ± 0.04	0.96 ± 0.07	0.96 ± 0.04	0.96 ± 0.03	0.99 ± 0.01	2	2
Ensemble + Coudray et al.^9^	PKM2+Ang‐2	0.74 ± 0.14	0.78 ± 0.18	0.86 ± 0.11	0.80 ± 0.08	0.80 ± 0.14	5	8
Proposed Method	PKM2+Ang‐2	**0.99** ± **0.03**	**0.99** ± **0.02**	**0.99** ± **0.02**	**0.99** ± **0.02**	**1.00** ± **0**	1	1

*Note*: In the last group of Table 1, the first three rows represent the result of each model trained based on the concatenation of the images of the two markers, while the fourth to sixth row represent the result of ensemble decisions from two separate models, and each model is trained independently from the images of individual markers.

In conclusion, these findings suggest that the proposed ensemble model could assist treatment planning for personalized medicines and may be used as a new potential biomarker system to predict the bevacizumab therapeutic effect. Further conclusion and discussion are in the supporting information (Section 4).

## CONFLICT OF INTEREST

There is no competing interest, which influences the work reported in this study.

## Supporting information

Supporting Information
