# Supplementary material for: Ensemble biomarkers for guiding anti‐angiogenesis therapy for ovarian cancer using deep learning
Source: Clin Transl Med. 2023 Jan 13;13(1):e1162. doi: 10.1002/ctm2.1162 (PMC9839833; doi:10.1002/ctm2.1162)
Supplement: Supplementary file 1 — Supporting Information [file CTM2-13-e1162-s001.docx]

**Ensemble biomarkers for guiding anti-angiogenesis therapy for ovarian cancer using deep learning**

Supporting Information

***Summary***

The main contribution of this study is to introduce a highly effective biomarker system for prediction of the therapeutic response in guiding ovarian cancer treatment. We develop an improved and interpretable annotation-free instance boosting deep learning ensemble approach and explore three different angiogenesis related potential biomarkers, i.e. VEGF, Ang-2 and PKM2. The proposed method and three recently published state-of-the art weakly supervised deep learning approaches [21], [22] [25] are evaluated on the new database. The experimental results show that the proposed ensemble model using both PKM2 and Ang-2 expression achieves excellent performance in bevacizumab therapeutic effect prediction, achieving a notably high F-score (1), accuracy (1), precision (1), recall (1) and AUC (1) in the first experiment with (66% for training and $34\%$ for testing) and a consistent notably high F-score ( $0.99\pm0.02)$, accuracy $(0.99\pm0.03)$, precision $(0.99\pm0.02)$, recall $(0.99\pm0.02)$ and AUC $(1.00\pm0)$ in the second experiment using 5 -fold cross validation. Moreover, Kaplan-Meier progression free survival (PFS) analysis shows that the proposed ensemble model is able to distinguish patients gaining positive therapeutic effects with low cancer recurrence from patients with disease progression after treatment $(p<0.001)$, and the Cox proportional hazards model analysis further confirms the above statement ( $p=0.012$ ). These findings suggest that the proposed ensemble model could assist treatment planning for personalized medicines and may be used as a new potential biomarker system to predict the bevacizumab therapeutic effect.

***Section 1. Related Works***

*1. Selection of Potential Biomarkers:* In this study, three angiogenesis related potential biomarkers are selected for investigation, including VEFG, Ang-2 and PKM2, for the following reasons. Firstly, VEGF and its signaling pathway plays an important part in the development of tumor pathological vascular networks and is often overexpressed in epithelial ovarian cancer (EOC) [1]. Bevacizumab neutralizes VEGF preventing activation of the VEGF receptor and therefore an attractive target for investigation. Secondly, Angiopoietin 2 (Ang-2) is selected as it is a well-established promoter of vascular leak, induced in high vascular permeability states such as sepsis [2] and has also been shown as a potential angiogenic biomarker to predict malignancy of ovarian neoplasm and poor prognosis in EOC [3]. Moreover, anti-angiogenics other than anti-VEGF agent for EOC such as targeting Ang-2 has also been developed [4]. Thirdly, the Pyruvate kinase isoform M2 (PKM2) is highly expressed in most cancer cells and has been extensively studied as a driver of oncogenic metabolism. Metabolic reprogramming is fundamental to cancer initiation and progression, in which PKM2 has been demonstrated to play a decisive role in aerobic glycolysis (Warburg effect) inducing angiogenesis and, ultimately, resulting in tumor metastasis [5] [6]. Considering the importance of angiogenetic mechanism of bevacizumab treatment, VEGF, Ang-2 and PMK2 are explored in this research as potential new targets to predict bevacizumab therapeutic effect in EOC patients.

*2. Weakly Supervised Learning in Computational Pathology:* Recent studies have investigated deep learning approaches in cancer diagnosis, prognosis and therapeutic response predictions using histopathological whole slide images [7]. Conventional deep learning algorithms for computational pathology however require either considerable amounts of manual annotations of regions of interest (ROI) of gigapixel whole-slide images (WSIs), which is laborious and often biased, or massive datasets of WSIs with slide-level labels and usually perform poor in domain adaptation and interpretability [8]. However, such sizable datasets are not only very expensive but also difficult to curate for precision oncology applications where only limited number of samples may exist or for clinical trials where it may be useful to predict outcome from a small cohort of patients.

Weakly supervised learning is a form of learning where unannotated images with only image-level labels are used in training [9]. Campanella et al. [21] introduced a weakly supervised multiple instance learning-based deep learning approach that uses only the reported diagnoses as labels for training without pixel-wise manual annotations, and three slide diagnosis systems were built and tested for prostate cancer, basal cell carcinoma and breast cancer metastases, respectively, for classification of slides into three categories, including benigh/negative, atypical/other/suspicious and carcinoma/positive. Coudray et al. [22] trained an Inception V3 network on whole slide images for classification of lung histopathological slides into adenocarcinoma, squamous cell carcinoma and normal types. Chen et al. [10] also built a deep learning system for classification of lung histopathological slides into two types, i.e. adenocarcinoma and squamous cell carcinoma. In 2022, Wang et al. published a histopathological whole slide image dataset for classification of treatment effectiveness to ovarian cancer [11], further constructed a database of immunohistochemical tissue microarrays of AIM2, C3, C5 and NLRP3 and developed a weakly supervised learning model AIM2-DL, which obtains F-score $0.87\pm0.06$ using 5fold cross validation in classification of patients gaining positive therapeutic effects from patients with disease progression after treatment [12].

***Section2. Materials and Methods***

***Section 2-1. Materials***

The clinicopathologic characteristics of patients were recorded by the data managers of the Gynecologic Oncology Center. Regarding the subject demographics, the age of patients ranges from 23 to 79 years old (mean 58.75). Pre- and post-treatment serum CA125 concentrations, histologic subtype, and recurrence status were recorded. Measurement of disease with CT imaging is the most reliable measurement of tumor recurrence [13], and CA125 is an alternative reliable marker with respect to the extended bevacizumab treatment [14]. However, no predictive biomarker has been identified for personalized use of bevacizumab in EOC [15]. We attempt to identify angiogenesis related biomarkers of bevacizumab therapeutic effect based on VEGF, Ang-2 and PKM2 immunostaining images in tissue microarray (TMA) of EOC and peritoneal serous papillary carcinoma (PSPC). Each patient in the tissue microarray has a complete medical records about sensitive or resistant to bevacizumab therapy. A tumor, which is resistant to bevacizumab therapy, is defined as a measurable regrowth more than 2 cm of the tumor (based on CT/PET imaging) or high levels of serum CA125 concentration (more than 2 times of the upper limit 35 U/mL) during or within six months of bevacizumab treatment. A tumor, which is sensitive to bevacizumab therapy, is defined as no measurable regrowth of the tumor (based on CT/PET imaging) or low level of serum CA-125 concentration (below of the upper limit 35 U/mL) during or within six months of bevacizumab treatment. With regard to the class distribution of the collected database, a well-balanced data set is ensured, and 412 tissue cores (57.2%) are associated with effective Bevacizumab treatment outcomes whereas 308 cores $(42.8\%)$ are associated with invalid treatment outcomes.

Tissues from bevacizumab treated EOC and PSPC patients embedded in Paraffin wax. Two pathologists screened the histological sections and selected areas of representative tumor cells, and one tissue core ( $2\text{ }\mathrm{mm}$ in diameter) was then taken from each of the representative tumor samples and placed in a new recipient paraffin block for immunohistochemistry staining. The TMA sections were dewaxed in xylene, rehydrated in alcohol, and immersed in 3% hydrogen peroxide for $10mins$ to suppress the activity of endogenous peroxidase. Antigen retrieval was performed by heating each section to ${100}^{\circ}C$ for 30 mins in $0.01M$ sodium citrate buffer ( $\mathrm{pH}$ 6.0). After three 5-min rinses in phosphate-buffered saline (PBS), the sections were incubated for $1\text{ }h$ at room temperature with antibody of anti-VEGF (1:50) (Abcam, cat#ab2350, Cambridge, UK), anti-Ang-2 (1:100) (Abcam, cat#ab56301, Cambridge, UK), anti-PKM2 (1: 200) (Cell Signaling Technology, #4053, Massachusetts, USA) in PBS. The sections were washed three times (5 mins each wash) in PBS, followed by incubation with horseradish peroxidase-labeled immunoglobulin (Dako, Carpinteria, CA, USA) for $1\text{ }h$ at room temperature. The sections were washed three times again, and the peroxidase activity was visualized using a solution of diaminobenzidine (DAB) at room temperature. Slides were counterstained with hematoxylin. Control samples were processed similarly, with the exception of the omission of the primary antibody. The WSIs were acquired with a digital slide scanner (Leica AT Turbo) with a 20x objective lens.

***Section 2-2. Methods***

With deployment in clinical practice, the development of medical decision support systems has been impeded by the need of sizable amounts of training data. However, large datasets are difficult to build especially in precision oncology applications, which require collecting data and tracking patients' responses for years from limited patients, who have been diagnosed with the cancer type of interest and received targeting drugs or treatments. A common solution to the problem of limited data is data augmentation, which increases the diversity and amount of data by adding modified copies or newly created synthetic data of existing data and acts as a regularizer to help reduce overfitting when training an artificial intelligence model [16]. Another solution to the problem of limited data in computational pathology as introduced by Coudray et al. [22] is to divide each instance such as WSI to multiple patch units for greatly increasing the number of training samples and assign the same label to every patch for patch-wise training. In other words, if there are $N$ WSI instances to train originally, after splitting each instance into $K$ patches, the number of instances to train is dramatically increased to $N\times K;K$ is usually very large as a WSI usually contains more than 10,000 patches, i.e. $K>10,000$. This patch-wise training approach however suffers from noisy training data and is not applicable to problems that contain limited tumor cells [17].

To deal with noisy training data issue caused by patchwise training, Campanella et al. [21] presented a two stage approach, in which the first stage MIL model produces the tumor probability of each patch, ranks the patches according to the probability and then selects only the top $s$ patches to represent each training instance for learning the second stage RNN classifier where $s=10$ in their implementation. In other words, the number of instances for training the classifier is increased to $N\times10$, which is much smaller than $N\times K$ but the training data quality is assured by the first stage MIL model. However, there are two disadvantages of Campanella et al.'s approach [21]. Firstly, the database for training the second stage RNN classifier might not be large enough to train a reliable classifier with low generalization error for unseen testing data. Secondly, as shown in Fig. S2, the first stage MIL model might select patches without or with only a few tumor tissues, which consequently influences the performance of the final second stage RNN classifier, causing wrong model predictions in patients' treatment responses.

In our previous work [12], patch-wise weakly supervised learning with boosted data augmentation was devised to train the first stage model at $20X$ magnification level to locate tumor-like tissues, and afterwards patches of tumor-like tissues of each tissue core are integrated and down-sampled to a $512\times$ 512 patch as the core-representative for training the second stage classifier, which is trained using $N$ core-representative patches. This methodology, however, throws away a lot of valuable high resolution information and trains a classifier with a small number of instances, which may suffer from overfitting and the capability of the classifier could be constrained due to limited number of training instances and limited information from down-sampled data.

***Proposed Interpretable Annotation-Free Instance Boosting DL Ensemble***

To overcome the aforementioned issues, we develop an interpretable annotation-free instance boosting deep learning ensemble approach, which consists of a patch-wise instance boosting strategy to not only enlarge the database but also utilize high resolution information for training individual classifiers, a weakly supervised tumor selection model to locate tumor-like tissues, a data cleaning and validation module for producing a large, cleaned and qualified training database, a treatment effectiveness DL classification model, an attention scoring module for computing the importance of individual tiles and an ensemble framework to integrate multiple classifiers and produce the final decision. One of the contributions of this study is to develop an effective and efficient precision oncology system for prediction of therapeutic effect on ovarian cancer patients while utilizing as few manual annotations as possible. The general framework is illustrated in Fig. S1. Firstly, a weakly supervised Modified FCN is applied to fetch the tumor-like tissues, and a deconvolution layer is utilized for data cleaning, following a concatenate layer to aggregate the important information. Secondly, an attention scoring layer is devised to extract the representative patch(es) of a core with the most important information. Finally, the Inception V3 architecture is used to train a treatment response classification model.

***1. Proposed Al Training Strategy: Instance Boosting, Data Cleaning and Validation:*** In this study, we devise an effective AI training strategy to construct a larger, clean and high resolution training database in order to produce a robust and effective treatment response prediction model. Fig. 1 in main body presents the workflow for training individual treatment effectiveness classifiers of the proposed method. Patch-wise training is adopted to utilize high resolution data and increase the training database. To ensure the quality of the training data, we devise cleaning and validation modules, which collect a much larger and cleaned training database for building the final classifier.

Given tissue microarray WSIs in multi-resolution pyramid data structure $\left\{ Q_{l} \right\}_{l=0}^{L}$, a tissue core detection model $\Psi_{\text{cores}\text{ }}$, which is built with Cascade R-CNN [18], speedily locates $N$ tissue core instances $\left\{ \mathbf{b}_{\epsilon}^{d} \right\}_{d=1}^{N}$ in the low resolution $Q_{\xi}$.

$$\left\{ \mathbf{b}_{\epsilon}^{d} \right\}_{d=1}^{N}=\Psi_{\text{cores}\text{ }}\left( Q_{\epsilon} \right)$$

Then, forward-map to the high magnification level $Q_{0}$ to acquire high resolution core instance data $\left\{ \mathbf{b}_{0}^{d} \right\}_{d=1}^{N}$, which are split to $512\times512$ patch unit instances$\left\{ \mathbf{u}_{0}^{i} \right\}_{i=1}^{N^{'}}\mid N^{'}=\sum^{1\ldots N} M^{d}$where every core could be divided into $M^{d}$ patches. Hence, the quantity of training instances could be boosted from $N$ to $\sum^{1\ldots N} M^{d}$ as shown in Fig. 1 (b.i).

Next, a weakly supervised tumor-like tissue segmentation model $\Psi_{\text{tumor}\text{ }}$ from our previous work [12] is applied to $\left\{ \mathbf{u}_{0}^{i} \right\}_{i=1}^{N^{'}}$ to identify important information (see Fig. 1 (b.ii)), producing the pixel probabilities $\left\{ \left( p_{0}^{i}(x,y) \right)^{k} \right\}$.

$$\left( p_{0}^{i}(x,y) \right)^{k}=\Psi_{\text{tumor}\text{ }}\left( u_{0}^{i}(x,y) \right)$$

where $k\in\{0,\ldots,K\};k=0,1,2$ represents the background, non-tumor-like tissue and tumor-like tissue, respectively.

A data cleaning module formulated in equation 3 extracts the tumor-like tissue information and suppresses the rest of the information, producing cleaned data $\left\{ c_{0}^{i}(x,y) \right\}_{i=1}^{N^{'}}$ (see Fig. 1 (b.iii)).

$$c_{0}^{i}(x,y)=\left\{ \begin{matrix} u_{0}^{i}(x,y) & ,arg\max_{k} \left( p_{0}^{i}(x,y) \right)^{k}>1 \\ \emptyset& ,\text{ }\text{otherwise}\text{ } \end{matrix} \right.$$

To assure the training data quality to contain adequate important information, a data validation module is built to ensure that each patch instance $\mathbf{c}_{0}^{i}$ contains a minimum $\alpha$ level of information where $\alpha$ is set as $0.05$ in this study (see Fig. 1(b.iii)). The data validation discards instances full of useless information to avoid confusion or distraction in AI training, producing a large, clean and high quality training database $\left\{ \mathbf{q}_{0}^{i} \right\}_{i=1}^{N^{''}};N^{''}=\sum_{d=1}^{N} M^{d}-V$ and $V$ denotes the number of instances discarded by the data validation module.

$$\mathbf{q}_{0}^{i}=\left\{ \begin{matrix} \mathbf{c}_{0}^{i} & ,\frac{\left| \mathbf{c}_{0}^{i} \right|}{\left| \mathbf{u}_{0}^{i} \right|}>\alpha\\ \emptyset& ,\text{ }\text{otherwise}\text{ } \end{matrix} \right.$$

The data $\left\{ q_{0}^{i}(x,y) \right\}_{i=1}^{N^{''}}$ is then used to train a treatment response classification model $\Psi_{\text{classifier}\text{ }}$ based on an Inception V3 network [19] with dimensional reduction and parallel structures of the Inception modules, as illustrated in Fig. 1(b. iv-v). Table S1 compares the data sampling strategies for training the final decision model among the benchmark approaches and the proposed method. Overall, there are three major advantages of the proposed AI training approach in comparison. That is the proposed AI training method trains a final decision classifier based on a training database, which is large, cleaned and with detailed important information at high magnification level.

***2. Decision Inference Strategy: Data Cleaning, Attention Scoring and Ensemble Decision:*** Fig. 2 in main body presents the workflow in decision inference of the proposed ensemble model. Firstly, each patient's core instance $\left\{ \mathbf{b}_{\epsilon}^{d} \right\}_{d=1}^{N}$ in the low resolution $Q_{\xi}$ is rapidly located by the core detector $\Psi_{\text{cores}\text{ }}$, fetching high magnification data $\left\{ \mathbf{b}_{0}^{d} \right\}_{d=1}^{N}$, which is then cleaned by the data cleaning module as described in the previous section, producing cleaned patch instances $\left\{ \mathbf{c}_{0}^{d,j} \right\}_{j=1\ldots M^{d}}$ at high magnification level. Next, we devise an attention scoring function as formulated below to extract the representative patch $\gamma^{d}$ of the $d$-th core with the richest important information highlighted by $\Psi_{\text{tumor}\text{ }}$.

$$\gamma^{d}=\underset{j}{argm}\left| \mathbf{c}_{0}^{d,j} \right|$$

For each kind of protein expression data with the $z$-th antibody, the probability to be the prediction class $o$ is obtained using the treatment effective decision model $\Psi_{\text{classifier}\text{ }}^{z}$.

$$\left\{ P_{z}^{d} \right\}^{o}=\Psi_{\text{classifier}\text{ }}^{z}\left( \gamma_{z}^{d} \right)$$

where $o\in\{0\ldots O\};o=0$ represents invalid; $o=1$ represents effective in this study.

The final prediction $\omega_{z}^{d}$ of $d$-th core using $z$-th protein expression data is formulated as follows.

$$\omega_{z}^{d}=\underset{o}{argm}\left\{ P_{z}^{d} \right\}^{o}$$

The final ensemble decision $\Omega^{d}$ is computed by integrating the decisions of individual classifiers using various protein expression data as follows.

$$\Omega^{d}=\underset{o}{argm}\sum_{z} \left\{ P_{z}^{d} \right\}^{o}$$

***Section 3. Result***

To ensure fair comparison, all benchmark methods were tested with the recommended setting-ups according to the associated publications / GitHub sources, and all models are trained with 20,000 maximum iterations.

***A. Comparison of Model Intermediate Results***

To demonstrate the interpretability of the proposed approach, apart from evaluation on the final model prediction results, we further examine the intermediate data extraction outputs of the proposed method with the best performing benchmark approach, i.e. Campanella et al. [21]. The intermediate results are important as they are the data of interests of a model and are utilized to render the final model decision by the second stage classification model. Fig. S2 compares the intermediate data extraction results of two tissue core samples by the proposed method (see Fig. S2 $(a,c)$ ) and Campanella et al. [21] (see Fig. S2 (b, d)). The results show that the proposed method selects a tile with abundant tumor information (Fig. S2 (a2, c2)) and suppresses non-tumor-like information, producing cleaned data with rich tumor data (Fig. S2 $(a4,c4)$ ) as inputs for the stage 2 model to perform final model prediction. In comparison, Campanella et al.'s approach [21] chooses 10 tiles as inputs for the stage 2 model to produce final model prediction. However, in our experiments, some selected tiles may contain few or even no tumor tissue. In Fig. S2 (b) sample, most of the tiles do no contain tumors, and in Fig. S2 (d) $50\%$ of the tiles contain few or no tumor tissue, which might be the problem causing wrong prediction outcomes in treatment response.

***B. Statistical Analysis***

Investigation of how treatments influence disease progression during survivorship is commonly performed with two statistical analyses, i.e. the univariate Kaplan-Meier (K-M) survival analysis and the multivariate Cox proportional hazards regression analysis [20], which have been adopted in this study. All statistical analyses were performed using SPSS software [21].

***1. Univariate Kaplan-Meier Survival Analysis:*** In this study, the Kaplan-Meier survival analysis is performed with respect to both progression free survival time and overall survival time using the log-rank test to further verify the performance of the proposed models. Patients are categorized into two groups based on the model prediction outcomes (0: invalid, 1: effective), and the Kaplan-Meier curves show what the probability of an event, such as disease progression or survival, are at a certain time interval. Fig 3(b) shows that there are very strong evidences that the proposed ensemble using both PKM2 and Ang-2 and the proposed models using PKM2 or Ang-2 are able to identify patients gaining positive therapeutic effects with low cancer recurrence in the progression free survival time with high statistical significance $(p<0.001)$.

***2. Multivariate Cox Proportional Hazards Regression Model Analysis:*** A multivariate cox proportional hazards regression model analysis is performed to further assess and relate several risk factors simultaneously to progression free survival time. The risk factors include the proposed ensemble model prediction and possible clinical attributes such as a patient's age, BMI, number of treatments, cancer stage (FIGO), histology, surgery type and therapy type. Table $S2$ confirms that the proposed ensemble model is effective for patient selection with statistical significance ( $p=0.012)$, and $(HR=0.23)$ indicates that patients who are predicted with effective treatment responses are $0.23$ times as likely to get cancer recurrence, compared to patients predicted with invalid responses.

***Section 4. CONCLUSION AND DISCUSSION***

Here we show that DL can predict bevacizumab therapeutic effect on patients with EOC and PSPC directly from immunostained TMA WSIs, which is easily performed. A humanized anti-VEGF monoclonal antibody, is the most widely studied anti-angiogenesis agent both across tumor types and specifically in EOC [22]. Therapeutic resistance and escape have become practical limitations. A number of blood or tissue biomarkers have been postulated to predict therapeutic effect to anti-angiogenesis agents, but convincing clinical predictive biomarkers for bevacizumab efficacy remains elusive [23]. Given the cost, potential for toxicity, and finding that only a subset of patients will benefit from bevacizumab, patients who receive bevacizumab treatment should be carefully selected. To the authors' best knowledge, this is the first precision oncology framework, which could produce near-to perfect prediction of the therapeutic outcome of EOC and PSPC patients to assist treatment planning for personalized medicines and may be used as a new potential biomarker system to predict the bevacizumab therapeutic effect.

Many tumor cells have elevated rates of glucose uptake but reduced rates of oxidative phosphorylation. This persistence of high lactate production by tumor cells in the presence of oxygen, known as aerobic glycolysis or "Warburg effect" [24]. Glycolysis and mitochondrial fission and fusion play a pivotal role in angiogenesis [25]. Pyruvate kinase $(PK)$ catalyzes the third and last rate-limiting step in glycolysis. The PKM gene encodes PKM1 and PKM2 through alternative splicing. Cancer cells almost invariably express the PKM2 isoform [24] [26]. PKM 2 and NF- $\kappa$ B complexes subsequently augment VEGF transcription. Altered expression of glucose uptake and aerobic glycolysis occurs in cancer tissues may be play an important role of anti-VEGF cancer therapy. In proliferating endothelial cells, PKM2 is required to suppress p53 and maintain cell cycle progression [27]. PKM2 was recently reported to have a central role in the metabolic reprogramming of cancer cells as well as participating in cell cycle progression and gene transcription [28]. At the peak of the response phase of anti-angiogenesis therapy, tumors have regions of acute hypoxia [29]. Hypoxia-inducible transcription factor is upregulated in response to hypoxia and is the central driver of the cascade of events that initiate metabolic reprogramming in cancer stem cells [30]. The increased VEGF is secreted extracellularly via exosomes, an event that is enhanced by the interaction of FOXM1 with VPS11, ultimately promoting tumor angiogenesis [31]. PKM2 also suppresses $NF-\kappa B$ and its downstream target, the vascular permeability factor Ang-2. [27].

We apply AI learning process in bevacizumab therapeutic effect data rather than focusing on traditional tumor pathological evaluation of tumor cell detection or subclassification. We have previously provided an automated weakly supervised DL framework for selection and guidance of bevacizumab targeted therapy in EOC and PSPC patients by analyzing immune-related biomarker, such as AIM2, NLRP3, C3 and C5, and the proposed-AIM2 model is demonstrated to be useful for bevacizumab therapeutic prediction [12]. Compared with our previous results, Ang-2 and PKM2 expression in EOC and PSPC described in this study may have greater potential to predict response to individualized anti-angiogenic therapy. However, it is still necessary to validate the PKM2 and Ang2 biomarkers for bevacizumab treatment prediction in patients with EOC and PSPC in larger population-based studies. Cancer has traditionally been examined by histopathology or cytopathology to confirm the presence of tumor cells within a patient sample. Convolutional neural network model training can be evaluated on TCGA H&E slides that accurately predict a range of actionable genetic alterations and genes for standard biomarkers expression [32]. DL offers the potential to infer important genomic features from readily available histopathology data, as well as disentangle the complex heterogeneity of tumor microenvironment to enable precision oncology. Meanwhile, DL algorithms can be used to learn patterns from the entire transcriptome, assigning cancers into clinically meaningful molecular subtypes that have treatment selection relevance [33]. Routinely used histopathological images have the potential to detect genomic signatures and may prove useful in the future to predict specific clinically meaningful molecular signatures without the need for tumor sequencing [30]. The proposed DL models could assist treatment planning for personalized medicines and potentially be used in patient selection of targeted drug therapy.

**REFERENCES**

[1] R. Siegel, D. Naishadham, and J. A, "Cancer statistics," CA: a cancer journal for clinicians, vol. 62, no. 1, pp. 10-29, 2012.

[2] A. du Bois, H. J. Lück, W. Meier, H. P. Adams, V. Möbus, S. Costa, T. Bauknecht, B. Richter, M. Warm, W. Schröder, S. Olbricht, U. Nitz, C. Jackisch, G. Emons, U. Wagner, W. Kuhn, J. Pfisterer, and A. G. O. O. C. S. Group, "A randomized clinical trial of cisplatin/paclitaxel versus carboplatin/paclitaxel as first-line treatment of ovarian cancer," Journal of the National Cancer Institute, vol. 95, no. 17, pp. 1320-1329, 2003.

[3] R. F. Ozols, M. Markman, and J. T. Thigpen, "Icon3 and chemotherapy for ovarian cancer," Lancet (London, England), vol. 360, no. 9350, pp. 2086-2088, 2002.

[4] R. K. Jain, "Antiangiogenesis strategies revisited: from starving tumors to alleviating hypoxia," Cancer cell, vol. 26, no. 5, pp. 605-622, 2014.

[5] N. M. Biel and D. W. Siemann, "Targeting the angiopoietin- $2/$ tie-2 axis in conjunction with vegf signal interference," Cancer letters, vol. 380 , no. 2, pp. 525-533, 2016.

[6] E. C. McClung and R. M. Wenham, "Profile of bevacizumab in the treatment of platinum-resistant ovarian cancer: current perspectives," International journal of women's health, vol. 8, pp. 59-75, 2016.

[7] R. A. Claussen, C. and L. Hanker, "Treatment of recurrent epithelial ovarian cancer," Geburtshilfe und Frauenheilkunde, vol. 80, no. 12, pp. 1195-1204, 2020.

[8] B. J. Monk, L. E. Minion, and R. L. Coleman, "Anti-angiogenic agents in ovarian cancer: past, present, and future," Annals of oncology : official journal of the European Society for Medical Oncology, vol. 27, no. 1, pp. i33-i39, 2016.

[9] J. Garcia, H. I. Hurwitz, A. B. Sandler, D. Miles, R. L. Coleman, R. Deurloo, and O. L. Chinot, "Bevacizumab (avastin®) in cancer treatment: A review of 15 years of clinical experience and future outlook," Cancer treatment reviews, vol. 86, p. 102017, 2020.

[10] R. Ren, J. Guo, J. Shi, Y. Tian, M. Li, and H. Kang, "Pkm2 regulates angiogenesis of vr-epcs through modulating glycolysis, mitochondrial fission, and fusion," Journal of cellular physiology, vol. 235, no. 9, pp. 6204-6217, 2020.

[11] Y. Feng, X. Li, J. Wang, X. Huang, L. Meng, and J. Huang, "Pyruvate kinase m2 ( $pkm2)$ improve symptoms of post-ischemic stroke depression by activating vegf to mediate the mapk/erk pathway," Nature reviews. Cancer, vol. 12 , no. 1,2022 .

[12] A. Zhang, L. Meng, Q. Wang, L. Xi, G. Chen, S. Wang, J. Zhou, Y. Lu, and D. Ma, "Enhanced in vitro invasiveness of ovarian cancer cells through up-regulation of vegf and induction of mmp-2," Oncology reports, vol. 15 , no. 4 , pp. 831-836, 2006.

[13] C. E. Haunschild and K. S. Tewari, “Bevacizumab use in the frontline, maintenance and recurrent settings for ovarian cancer,” Future oncology (London, England), vol. 16, no. 7, pp. 225–246.

[14] M. D. Dao, L. M. Alwan, H. J. Gray, H. K. Tamimi, B. A. Goff, and J. B. Liao, “Recurrence patterns after extended treatment with bevacizumab for ovarian, fallopian tube, and primary peritoneal cancers,” Gynecologic oncology), vol. 130, no. 2, pp. 295–299.

[15] G. C. Jayson, R. Kerbel, L. M. Ellis, and A. L. Harris, “Antiangiogenic

therapy in oncology: current status and future directions,”

[16] S. M. Parikh, "The angiopoietin-tie2 signaling axis in systemic inflammation," Journal of the American Society of Nephrology: JASN, vol. 28, no. 7, pp. 1973-1982, 2017.

[17] H. Sallinen, T. Heikura, S. Laidinen, V. M. Kosma, S. Heinonen, S. YläHerttuala, and M. Anttila, "Preoperative angiopoietin- 2 serum levels: a marker of malignant potential in ovarian neoplasms and poor prognosis in epithelial ovarian cancer," Nature biomedical engineering, vol. 20, no. 9 , pp. 1498-1505, 2010.

[18] K. Rohlenova, K. Veys, I. Miranda-Santos, K. De Bock, and P. Carmeliet, "Endothelial cell metabolism in health and disease," Trends in cell biology, vol. 28, no. 3, pp. 224-236, 2018.

[19] A. Pircher, L. Treps, N. Bodrug, and P. Carmeliet, "Endothelial cell metabolism: A novel player in atherosclerosis? basic principles and therapeutic opportunities," Atherosclerosis, vol. 253, pp. 247-257, 2016.

[20] C. L. Chen, W. H. Chen, C. C. andYu, S. H. Chen, Y. C. Chang, T. I. Hsu, M. Hsiao, C. Y. Yeh, and C. Y. Chen, "An annotation-free wholeslide training approach to pathological classification of lung cancer types using deep learning," Nature communications, vol. 12, no. 1, p. 1193, 2021.

[21] G. Campanella, M. G. Hanna, L. Geneslaw, A. Miraflor, V. W. K. Silva, K. J. Busam, E. Brogi, V. E. Reuter, D. S. Klimstra, and T. J. Fuchs, "Clinical-grade computational pathology using weakly supervised deep learning on whole slide images," Nature medicine, vol. 25 , no. 8, pp. 1301-1309, 2019.

[22] N. Coudray, P. S. Ocampo, T. Sakellaropoulos, N. Narula, M. Snuderl, D. Fenyö, A. L. Moreira, N. Razavian, and A. Tsirigos, "Classification and mutation prediction from non-small cell lung cancer histopathology images using deep learning," Nature Medicine, vol. 24, no. 10, pp. 1559 1567, Sep. 2018.

[23] J. Kim, K. C. Kim, H. J and, J. H. Lee, K. W. Kim, Y. M. Park, H. W. Kim, S. Y. Ki, Y. M. Kim, and W. H. Kim, "Weakly-supervised deep learning for ultrasound diagnosis of breast cancer," Scientific reports, vol. 11 , no. 1, p. 24382,2021 .

[24] C. W. Wang, C. C. Chang, M. A. Khalil, Y. J. Lin, Y. A. Liou, P. C. Hsu, Y. C. Lee, C. H. Wang, and T. K. Chao, "Histopathological whole slide image dataset for classification of treatment effectiveness to ovarian cancer," Scientific data, vol. 9, no. 1, p. $25,2022$.

[25] C. W. Wang, Y. C. Lee, C. C. Chang, Y. J. Lin, Y. A. Liou, P. C. Hsu, C. C. Chang, A. K. Sai, C. H. Wang, and T. K. Chao, "A weakly supervised deep learning method for guiding ovarian cancer treatment and identifying an effective biomarker," Cancers, vol. 14, no. 7, p. 1651, 2022.

[26] S. Tabe-Bordbar, A. Emad, S. D. Zhao, and S. Sinha, "A closer look at cross-validation for assessing the accuracy of gene regulatory networks and models," Nature biomedical engineering, vol. 8, no. 1, p. 6620 , 2018.

[27] C. W. Wang, S. C. Huang, Y. C. Lee, Y. J. Shen, S. I. Meng, and J. L. Gaol, "Deep learning for bone marrow cell detection and classification on whole-slide images," Medical image analysis, vol. 75, p. 102270, 2022.

[28] W. N. Dudley, R. Wickham, and N. Coombs, "An introduction to survival statistics: Kaplan-meier analysis," Journal of the advanced practitioner in oncology, vol. 7, no. 1, p. 91, 2016.

[29] C. Shorten and T. Khoshgoftaar, "A survey on image data augmentation for deep learning," Journal of Big Data, vol. 6, p. 60, 2019.

[30] M. Y. Lu, D. Williamson, T. Y. Chen, R. J. Chen, M. Barbieri, and F. Mahmood, "Data-efficient and weakly supervised computational pathology on whole-slide images," Nature biomedical engineering, vol. 5, no. 6, pp. 555-570, 2019.

[31] Z. Cai and N. Vasconcelos, "Cascade r-cnn: high quality object detection and instance segmentation," IEEE Transactions on Pattern Analysis and Machine Intelligence, 2019.

[32] C. Szegedy, V. Vanhoucke, S. Ioffe, J. Shlens, and Z. Wojna, "Rethinking the inception architecture for computer vision," in Proceedings of the IEEE conference on computer vision and pattern recognition, 2016, pp. 2818-2826.

[33] SPSS Inc., "Spss for windows. rel.17.0.1." Chicago: SPSS Inc.

Table S0. (a) Baseline characteristics of data.

| Characteristics | N |
| --- | --- |
| Tissue Core | 720 |
| Age (mean, range) | (58.75, 23-79) |
| BMI (mean, range) | (23.52, 16.2-38.7) |
| Diagnosis (%) | |
| Papillary serous carcinoma | 464 (64.44) |
| Peritoneal serous papillary carcinoma | 72 (10) |
| Clear cell carcinoma | 76 (10.56) |
| Unclassified carcinoma | 68 (9.45) |
| Endometrioid carcinoma | 32 (4.44) |
| Mucinous carcinoma | 8 (1.11) |
| FIGO stage (%) | |
| I | 68 (9.45) |
| II | 52 (7.22) |
| III | 412 (57.22) |
| IV | 188 (26.11) |
| Surgery (%) | |
| Optimal debulking | 292 (40.56) |
| CRS+HIPEC | 232 (32.22) |
| Suboptimal debulking | 196 (27.22) |
| Bevacizumab treatment method (%) | |
| Concurrent bevacizumab therapy | 224 (31.11) |
| Concurrent + maintenance bevacizumab therapy | 88 (12.22) |
| Second-line bevacizumab therapy post recurrence | 344 (47.78) |
| Second-line + maintenance bevacizumab therapy | 64 (8.89) |
| Treatment effectiveness (%) | |
| Effective | 412 (57.2) |
| Invalid | 308 (42.8) |

(b) Data distribution for the 1st experiment.

|  |  | PKM2 | Ang-2 | VEGF | PKM2+Ang-2 | #Samples |
| --- | --- | --- | --- | --- | --- | --- |
| Training | Effective | 68 | 68 | 68 | 68 | 472(68%) |
|  | Invalid | 50 | 50 | 50 | 50 |  |
| Testing | Effective | 35 | 35 | 35 | 35 | 248(34%) |
|  | Invalid | 27 | 27 | 27 | 27 |  |
| Total | | 180 | 180 | 180 | 180 | 720 |

TABLE S1: Comparison on Data Sampling Strategy in AI Training for the Final Model Classifier.


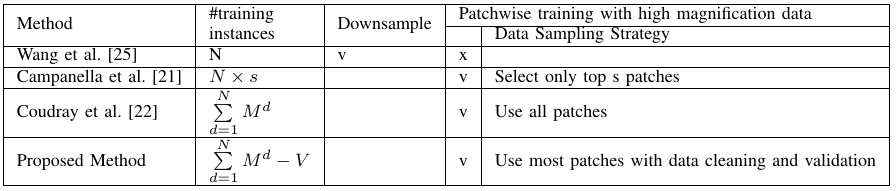


TABLE S2

Statistical Analysis: Multivariate Cox Proportional Hazards Regression Analysis in Cancer Recurrence.

|  | Adjusted HR¹ (95% C.I.) | P value |
| --- | --- | --- |
| Age | 0.98 (0.94-1.03) | 0.403 |
| BMI | 0.98 (0.89-1.08) | 0.694 |
| Number of bevacizumab used times | 0.99 (0.19-1.09) | 0.874 |
| FIGO² stage (III+IV vs. I+II)II | 2.65 (0.57-12.38) | 0.216 |
| Histology (others vs. serous) | 1.58 (0.55-4.52) | 0.395 |
| **Surgery** | | |
| CRS+HIPEC³ | 1.00 (reference) | reference |
| optimal | 0.87 (0.31-2.44) | 0.797 |
| suboptimal | 0.95 (0.32-2.85) | 0.928 |
| **Therapy** | | |
| Concurrent therapy | 1.00 (reference) | reference |
| Second-line therapy | 0.22 (0.04-1.24) | 0.086 |
| Maintenance therapy | 2.25 (0.71-7.10) | 0.165 |
| **DL model prediction** | | |
| Proposed Ensemble Method  (effective v.s. invalid) | 0.23 (0.08-0.72) | **0.012*** |

¹ HR = Hazard ratio; ² FIGO = International Federation of Gynecology and Obstetrics.

³ CRS+HIPEC = Cytoreductive surgery with hyperthermic intraperitoneal chemotherapy.

* Statistical significant (p<0.05)


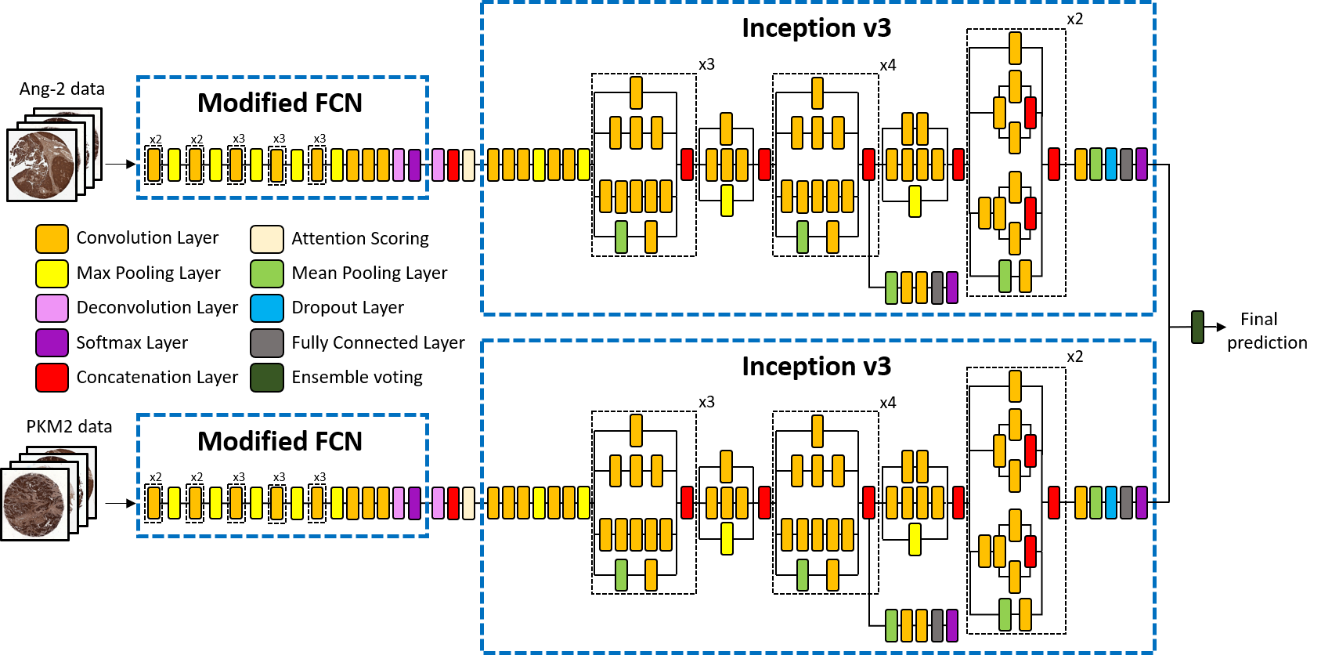
**Fig. S1: The general framework of the proposed method.**


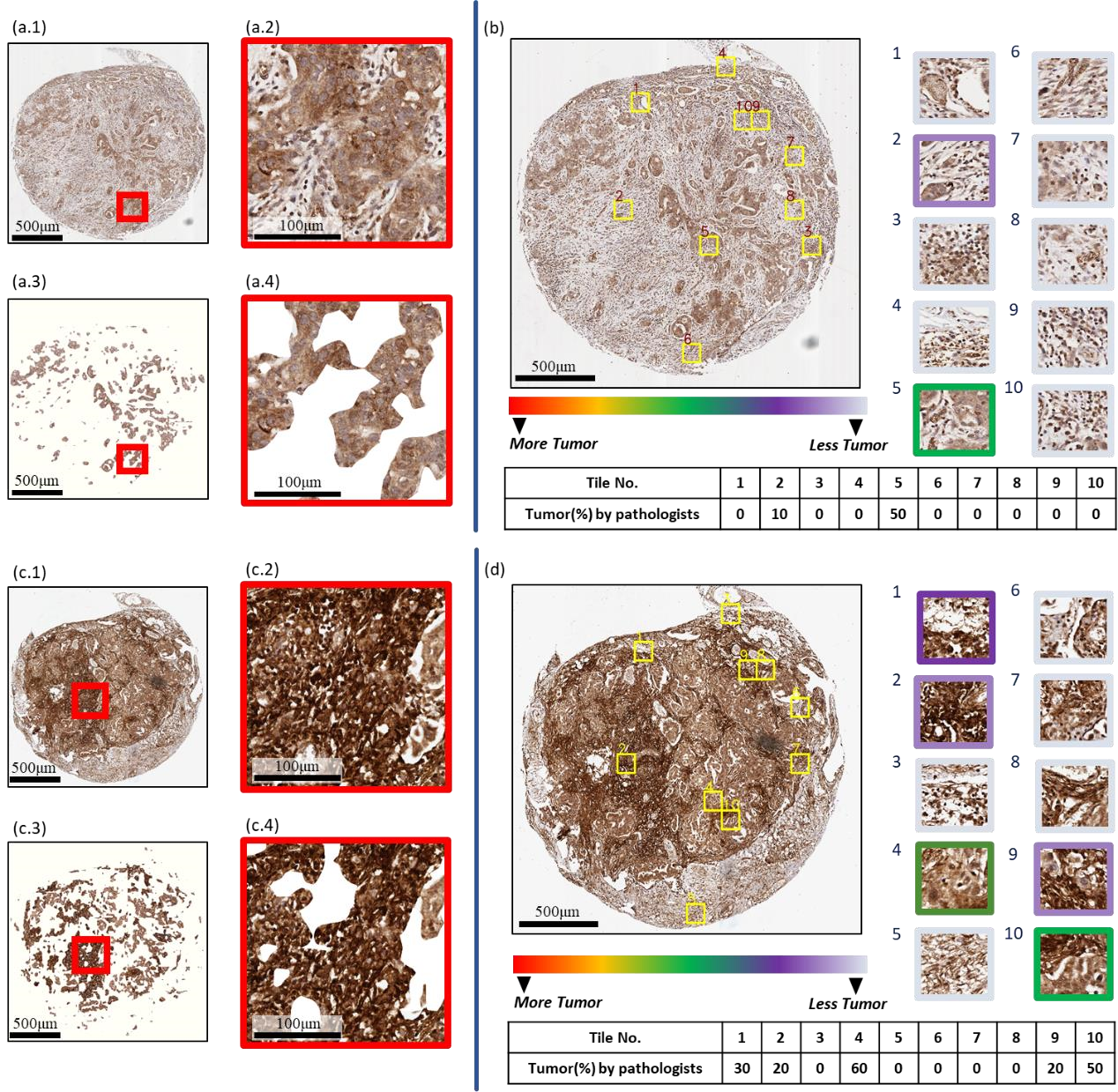


**Fig. S2: Comparison of the intermediate model outputs in extracting data of interests, which are inputs to the second stage classifier to render the final model decision, of the proposed method and the best performing benchmark approach, i.e. Campanella et al.** (a.1), (c.1) are the whole views of sample tissue cores with the region of interests highlighted in red by the proposed method, and (a.2), (c.2) are the associated high magnification views; (a.3), (c.3) are the whole views of data cleaning results by the proposed method, and (a.4), (c.4) are the associated high magnification views. (b,d) show the top 10 tiles extracted by Campanella et al.'s method with the percentage of tumor in each tile scored by pathologists. The results show that the proposed method fetches cleaned data with rich tumor information. In comparison, Campanella et al.'s approach sometimes fetches data of interests containing less or little tumor information, which might cause wrong final prediction outcomes.
